# The mechanism of fluorescence quenching of naphthalimide A/C leak detector by copper (II)

**DOI:** 10.1186/s13065-023-00987-2

**Published:** 2023-07-05

**Authors:** Ismail Badran, Najamus Sahar Riyaz

**Affiliations:** 1 Department of Chemistry, Faculty of Sciences, An-Najah National University, Nablus, Palestine; 2 College of Arts and Science, Qatar University, P.O. Box: 2713, Doha, Qatar

**Keywords:** Naphthalimide, Fluorescence quenching, Analytical, DFT, Dye, Cu^2+^

## Abstract

**Background:**

Fluorescence quenching is an interesting phenomenon with the potential to be applied across various fields. The mechanism is commonly used across analytical applications for monitoring the concentration of trace substances. Naphthalimide and its family of compounds are commonly used as fluorescent detectors. This work investigated an analytical technique through which naphthalimide-based dyes could be quantified. A commercial A/C leak detector was used as the dye and Cu^2+^ ions as the quencher. Experiments were also conducted to investigate the effect of temperature on quenching. To study the mechanism of quenching further, density functional theory (DFT) was used.

**Results:**

The method detection limit obtained in this work is 1.7 × 10^–6^ mol/L. The results from the quenching experiments demonstrated a pattern which fit a modified Stern–Volmer (SV) model, with an R^2^ value of 0.9886. From the experiments on the effect of temperature, a dynamic quenching behavior was observed given the emission spectra demonstrated an inverse relationship with temperature.

**Conclusions:**

The quenching of the commercial A/C dye by Cu^2+^ ions can be used to develop a rapid and sensitive detection method for metal ions such as Cu^2+^, and for future fabrication of chemosensors for Cu^2+^.

## Introduction

Fluorescent materials have exhibited great promise in different fields such as chemical sensing, displays, biological imaging, and quantum computing [1–4]. The importance of fluorescent stems from their high sensitivity, non-invasiveness, and high spatial and temporal resolution [5–7]. One of the most common applications of fluorescent chemicals is as leak-detecting dyes. Due to their rapid response to UV light, such dyes are widely used in automobile repair shops, the oil and gas industry, research labs, as well as in the air conditioning (A/C) industry [8, 9]. Although many of these dyes are claimed to be nontoxic and environmentally friendly, the long-term effects of fluorescent dyes and their fate is still being debated [10, 11]. In fact, little attention was devoted into the environmental impact of the fluorescent dyes’ overuse and their possible spillage into water systems. As a result, this work was motivated to understand their fluorescence behavior, and their interaction with metal ions, and determine whether such interaction results in a chemical or physical interaction with heavy metal ions. The study's findings could also be applied to the development of a quick and sensitive analytical method that employs fluorescence spectroscopy to quantify the concentration of metal ions when used as quenchers.

The use of fluorescence spectroscopic methods is considered one of the most precise and effective analytical techniques [5, 6]. While chromatographic techniques (e.g. HPLC, LCMS) are expensive and time-consuming, fluorometric analysis is straight forward, accurate, and requires little training [12, 13]. Fluorescence quenching is an interesting phenomena that is used in analytical analysis. Quenching is the process that decreases the fluorescence intensity of a sample [14]. For instance, Zhang et al., used rhodamine-110, a strong fluorescent dye, for accurate determination of trace nitrite ions in water samples [15]. In a previous study, [16] a fluorescence assay was developed for Curli proteins that exploits the fluorescent features of Congo Red. In addition to traditional organic dyes, smarter fluorescent materials such as carbon quantum dots and molecular organic frameworks are now being developed [13, 17–19].

**Scheme 1:**
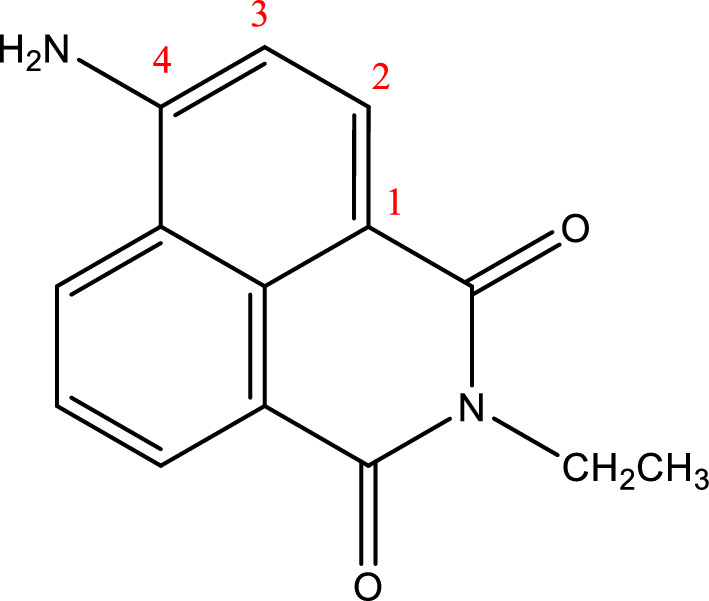
4-Amino-1,8-naphthalimide (CAS number 1742–95-6)

One of the most commonly used fluorescent materials is naphthalimide and its derivatives [20]. These fluorophores became attractive due to functionality at the 4th position of the naphthalimide ring [8, 20]. By adding either electron-withdrawing or donating groups, the electronic, optical, and photochemical properties of naphthalimides can be altered and thus optimized [20, 21]. For instance, by introducing the amino electron-donating group (Scheme 1), naphthalimide becomes more selective towards transition metal ions such as Co^2+^ and Cu^2+^ [5, 8, 20].

The first objective of this study is to comprehend the quenching mechanism of the naphthalimide derivative. A commercial A/C leak detector was used as a model dye, and copper (II) ions were used as a quencher. The results were fitted to different quenching models including that of Stern–Volmer (S-V), the sphere of action model, and the modified SV model. The effect of temperature on the quenching was also systematically studied to distinguish static from dynamic quenching. Usually called ‘collisional quenching’, dynamic quenching involves the diffusion of the quencher to the fluorophore during the life time of the excited state [14]. As a result, the fluorophore returns to its ground state without emission of a photon. Static quenching, on the other hand, assumes that the fluorophore and the quencher form a non-fluorescent pair [12, 14].

The interaction between Cu^2+^ ion and the naphthalimide was also studied using density functional theory (DFT), and the absorption spectra before and after the quenching were obtained using time-dependent density functional theory (TDDFT). The quantum theory of atoms in molecules (QTAIM) was used to comprehend the interaction between the Cu^2+^ ion and the naphthalimide. Given the current knowledge gap regarding the mechanism of naphthalimide quenching by Cu^2+^, we examined the results of the quenching models and proposed a mechanism of action that explains the quenching behavior of naphthalimide caused by the copper (II) ion. The mechanism was supported by the results of the DFT. TDDFT, and QTAIM calculations.

The second objective of this work is to develop an analytical technique to quantify the concentration of naphthalimide-based fluorescent dyes. This is due to the environmental concern surrounding the widespread use of such dyes [10, 22]. More importantly, the concentration of copper(II) themselves can be also determined in aqueous media, when used as quencher.

In the first part of this manuscript, we outline the results obtained from the experimental and theoretical studies. The results are discussed under the light of the DFT and QTAIM calculations in order to propose a quenching mechanism for naphthalimide-based fluorescent dye using Cu^2+^ as a quencher. Finally, the outcomes are used to provide a step-by-step procedure to quantify either A/C dye detector or Cu^2+^ ions in aqueous media.

## Experimental work

### Materials and instruments

A commercial A/C leak detector (Robinair 16241 Tracker Universal A/C Dye, Service Solutions US LLC, Minnesota, USA) was used in this work as a model fluorescent dye. The dye contains 4-amino-1,8-naphthalimide (CAS number 1742-95-6) as the main ingredient. Ethyl acetate (anhydrous, 99.8%) and copper(II) nitrate trihydrate (Cu(NO_3_)_2_.3H_2_O, ≥ 99.9% trace metals basis) were purchased from Sigma-Aldrich, Dorset, United Kingdom. All fluorometric measurements were done using a 1-cm quartz cell. Milli-Q® water was used to prepare all solutions. The study was done using a fluorescence spectrophotometer (RF-6000, Shimadzu, Maryland, USA) equipped with a temperature controller.

### Solutions preparation

The aromatic AC dye is not completely soluble in water, so it was dissolved in water/ethyl acetate mixture. A stock solution of 5.0 × 10^–3^ mol/L of the A/C dye (referred hereinafter by AC) was prepared in Milli-Q water/ethyl acetate. Similarly, a 1.0 × 10^–3^ mol/L of the Cu^2+^ quencher (referred hereinafter by Q) was also prepared and covered with aluminum foil. For the calibration curve, working solutions were prepared in the quartz cell by mixing different amounts of Q, ethyl acetate, and water to keep a total volume of 5.0 mL. This allows the formation of different concentrations in the range of 1.0–8.0 × 10^–4^ mol/L of the quencher but keeping a constant fluorophore concentration (F_0_ = 1.0 × 10^–3^ mol/L) under the same total volume. For the quenching study, different mixtures were prepared by altering the ratios of Q and AC keeping a total volume of 5.0 mL.

### Fluorometric measurements

The excitations and emission spectra for water and ethyl acetate were recorded to ensure the absence of any interference. The optimum excitation wavelength of AC was obtained by screening the region between 220 and 320 nm, and the best excitation was found to be at 280 nm, which was used throughout this work. The excitation and emission bandwidths were fixed at a slit width of 5 nm and a speed of 6000 nm/min. For a typical quenching experiment, the desired volume of AC was transferred to the quartz tube using a micropipette along with definite amounts of ethyl acetate and water. Then a sample of the quencher was added to a total volume of 5.0 mL and the emission spectrum was recorded immediately. For the temperature study, all solutions were thermostatted for at least 5 min before performing the experiments. The study was done with fixed concentrations of both AC and the quencher. The temperature was controlled throughout the experiment and no temperature fluctuations were observed. The cuvettes were covered during all experiments to prevent evaporation.

### Theoretical calculations

For the sake of this theoretical study, 4-amino-1,8-naphthalimide was used, as it is the major ingredient in the commercial AC dye sample. All species in this work were first optimized using the B97D3 functional, [23, 24] and the 6–31 + g(d,p) basis set. The B97D3 functional developed by Grimme contains the necessary dispersion parameters required for the study. The level of theory used in this work has demonstrated excellent performance for similar systems [25–28]. The optimization was done using implicit solvation with ethyl acetate by including the scrf = (solvent = ethylethanoate) keyword in Gaussain. Frequency calculations were requested after each optimization to ensure the absence of any imaginary frequencies. Restricted Hartree–Fock were considered for closed-shell species, while unrestricted calculations were requested for the open shell systems, such as that of Cu^2+^ ion. The TDDFT calculations were done under the same level of theory of the DFT calculations. The DFT, and TDDFT calculations were done using Gaussian 16 Rev C.01 [29] and viewed using Gaussview [30]. The calculations of the quantum theory of atoms in molecules (QTAIM) were done starting from the optimized structures at B97-D/DZP level, and using ethyl acetate as a solvent. Scalar relativistic effects were treated with the ZORA approach [31, 32], and the frozen core approximation was set to none. All geometry optimizations were completed with an energy cut-off of 0.0005 Ha, and a gradient convergence of 0.005 Ha/Å. The QTAIM calculations were done using the ADF engine in Amsterdam Modeling Suite (AMS) 2022 [33–35]. Further details on the calculations used in this work were reported in previous publications [36–41].

## Results and discussion

### Calibration and method validation

**Fig. 1:**
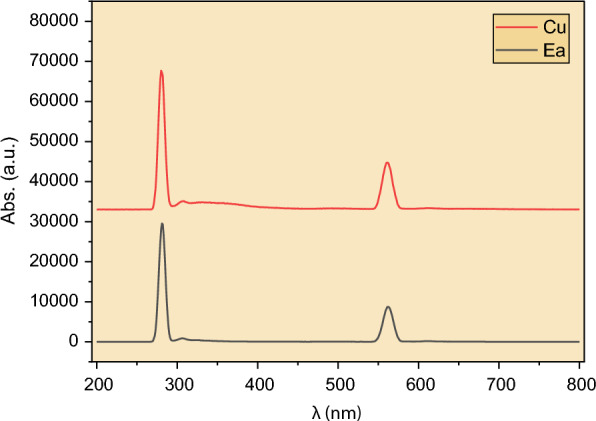
Room-temperature emission spectra for the water/ethyl acetate blank solution (black line), and 0.001 mol/L Cu^2+^ solution (red line). Excitation wavelength = 280 nm

Fluorescence spectrum of the water/ethyl acetate blank solution was recorded to ensure there were no interferences. A similar spectrum was also recorded for the Cu^2+^ solution, as shown in Fig. 1. As illustrated, the only peaks shown in the figure are the excitation peak at 280 nm and its duplicate peak at 560 nm. As a result, neither solution exhibits fluorescence in the 200–800 nm range.

**Fig. 2:**
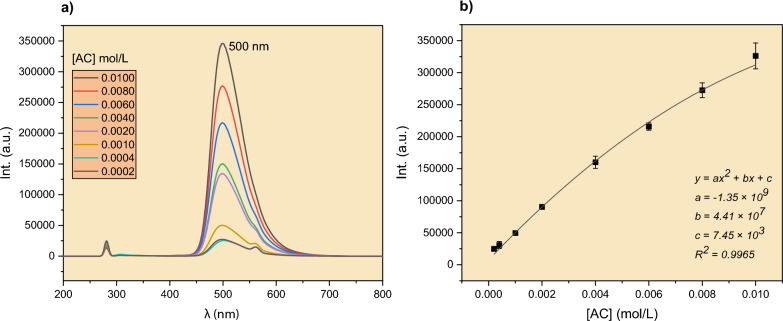
**a** Room-temperature emission spectra for AC with different concentrations in the range of 0.0002–0.0100 mol/L, **b** calibration curve for AC for the same concentration range at 25 °C. Excitation wavelength = 280 nm

The fluorescence emission spectra of the AC dye are shown in Fig. 2a. The spectra showed a maximum intensity centered at 500 nm in accordance with the green color of the dye. As shown in the figure, the intensity increased as the dye concentration increased from 0.0002 to 0.0100 mol/L. A calibration curve was then constructed (Fig. 2b) and found to follow the polynomial y = −1.35 × 10^9^x^2^ + 4.41 × 10^7^x + 7.45 × 10^3^. The model was then used to determine the AC concentrations throughout this work.

The spectrum of the blank solution was used to find the method detection limit (MDL), which is defined as the lowest concentration of analyte that can be measured with confidence [42–44]. MDL can be considered as three times the signal to noise ratio (3 × S/N) of the spectrum. The standard deviation of different measurements of the blank solution can be used to estimate the S/N value [44]. The MDL in terms of concentration units can then be determined from the slope and intercept of the calibration curve. Consequently, the MDL in this work is estimated to be 1.7 × 10^–6^ mol/L. The method quantitation limit (MQL), on the other hand, can be defined as the lowest concentration of an analyte that can be quantified using a given analytical procedure [42–44]. MQL can be calculated as three times the MDL, which equals be 5.1 × 10^–6^ mol/L. the sensitivity of the method was found from the slope of the linear fitting of the calibration curve, and it is equal to 3.8 × 10^7^ L/mol.

### The fluorescence quenching of the AC dye by copper (II) ions

**Fig. 3:**
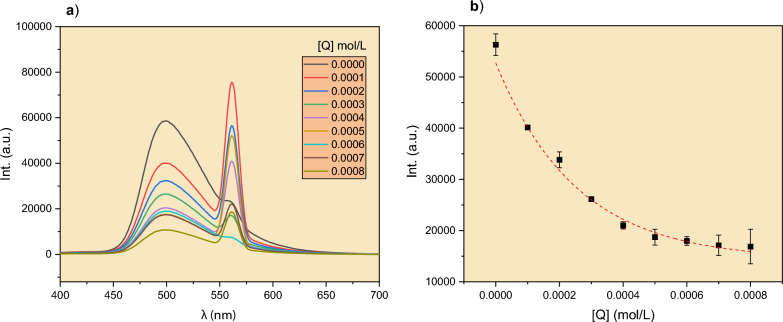
**a** Emission spectra of AC at 280 nm after being quenched by different concentrations of Cu^2+^ ions in the range of 0.0001–0.0008 mol/L. **b** The emission intensity of AC at 500 nm as a function of [Q]. Temp = 25 °C, [AC] = 0.001 mol/L. Error bars represent the standard deviation of three replicates. The peak at λ = 560 nm is the duplicate peak of the excitation peak at λ = 280 nm

The quenching of the AC dye was studied by adding different amounts of Cu^2+^ ions to the mixture while keeping a constant concentration of AC. Figure 3a shows the emission spectra of AC in the presence of the quencher. Clearly, as [Q] increased, the intensity of the emission at 500 nm decreased monotonically. Figure 3b depicts this decrease as a function of [Q]. The plot reaches a plateau with high concentrations of Cu^2+^ ions, indicating that the dye has been almost completely quenched.

**Fig. 4:**
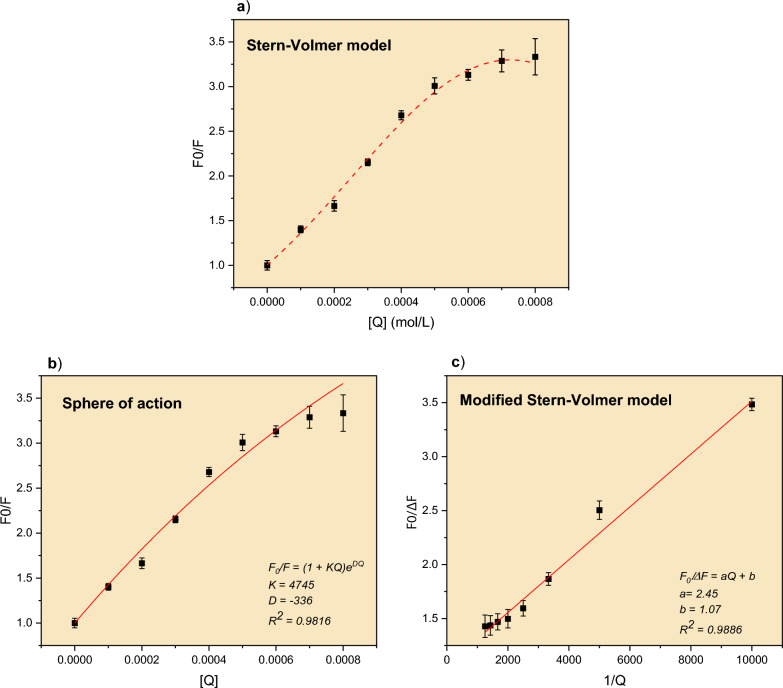
Fitting of the quenching data of AC by Cu^2+^ ions, **a** Stern–Volmer model, **b** sphere of action model, and **c** modified Stern–Volmer model. Temp = 25 °C, [AC] = 0.001 mol/L

In order to comprehend the mechanism of AC quenching by Cu^2+^ ions, the quenching data were first fitted to the Stern–Volmer (S-V) model given by the relationship [14]:

1 $$\frac{{F}_{0}}{F}=1+ {K}_{D}\left[Q\right]$$

where *F*_0_ and *F* are the fluorescence intensities in the absence and presence of quencher. Q is the quencher’s concentration, and *K*_D_ is the S-V quenching constant. If the quenching is known to be dynamic, the S-V constant will be represented by *K*_D_. In the case of static quenching, however, the constant will be denoted as *K*_S_. Figure 4a shows the S-V plot for the data obtained in this study. The figure shows a negative deviation (curvature towards the x-axis) from the expected linear behavior, indicating that the mechanism is more complicated. The data was then fitted to the mixed static and dymanic model given by [14]:

2 $$\frac{{F}_{0}}{F}=1+ {(K}_{D}+{K}_{S})\left[Q\right]+{K}_{D}{K}_{S}{\left[Q\right]}^{2}$$

where a plot of $\left(\frac{{F}_{0}}{F}-1\right)/Q$ vs. [*Q*] should give a straight line with slope = *K*_D_*K*_S_, and an intercept of = *K*_D_ + *K*_S._. Fitting the data (not shown) according to Eq. 2 has failed and therefore rejected. Thus, we attempted the sphere of action model [14]. Such a model suggests that the fluorophore and quencher don’t form a ground-state complex due to weak interaction. Instead, the quencher is positioned next to the fluorophore at the moment of excitation [14]. The sphere of action model is given by the equation [14]:

3 $$\frac{{F}_{0}}{F}=1+ {K}_{D}[Q]{e}^{\left[Q\right]VN/1000}$$

where *V* the volume of the complex sphere, and *N* is Avogadro’s number. The fitting result is shown in Fig. 4b. Despite the fact that the fitting is successful, with an adjusted R^2^ value of 0.9781, it is unacceptable due to the negative exponent. This result is rejected because no meaningful sphere volume can be obtained.

The failure of the above models suggests that the quenching of AC by Cu^2+^ ions may follow a more complicated mechanism. To explain such quenching behavior, a modified S-V model was previously proposed [14], which assumes that fluorophores are divided into two populations with different access to the quencher. In this model, the total fluorescence of the fluorophore is given by [14]:

4 $${F}_{0}={F}_{0a}+{F}_{0b}$$

where *F*_0a_ and *F*_0b_ are the accessible and inaccessible (buried) populations. The model suggests that the difference between *F*_0_ and the *F* (i.e., Δ*F*) is related to the quencher concentration [*Q*] by the following equation: [14]

5 $$\frac{{F}_{0}}{\Delta F}=\frac{1}{{f}_{a}{K}_{a}[Q]}+ \frac{1}{{f}_{a}}$$

where *f*_a_ is the fraction of the initial fluorescence that is accessible to the quencher. Therefore, a plot of *F*_0_/Δ*F* vs. 1/[*Q*] should give a straight line with a slope = 1/*f*_a_*K*_a_ and an intercept = 1/*f*_a_. Such plot is shown in Fig. 4c. The adjusted R^2^ value for the linear fitting is 0.9886, indicating a successful fitting. The value of *f*_a_ and *K*_a_ were found to equal to 0.9342 and 4329 L/mol, respectively. The near-unity value of *f*_a_ suggests that most of AC population was indeed accessible to the quencher over the course of their interaction.

### Effect of temperature on the quenching

**Fig. 5:**
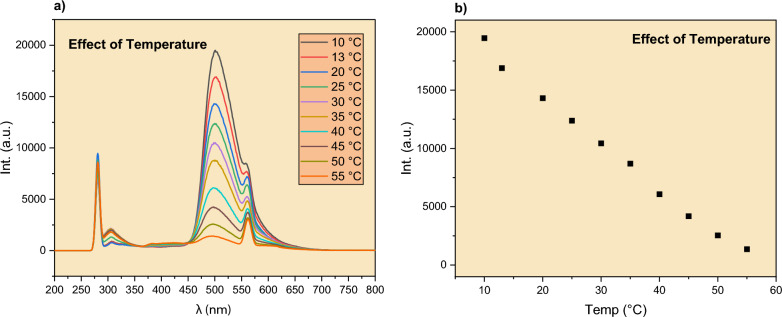
Effect of temperature on the quenching of AC by Cu^2+^ ions, **a** emission spectra at different temperatures in the range of 10–55 °C. **b** The emission intensity of AC at 500 nm as a function of temperature. [AC] = 0.001 mol/L. [Q]: 0.0003 mol/L

We demonstrated that the quenching of AC by Cu^2+^ ions follow a modified S-V model. The model, however, does not specify whether the quenching was dynamic or static. To distinguish between the two, we performed quenching experiments at different temperatures in the range of 10–55 °C. As shown in Fig. 5a, as the solution temperature increased, the emission spectra of AC in the presence of Cu^2+^ ions decreased monotonically. This is also reflected in the relationship between the AC intensity (λ_max_ = 500 nm) and temperature, as illustrated in Fig. 5b. This is typical dynamic quenching behavior, in which higher temperatures cause faster diffusion, resulting in a higher rate of collisions with naphthalimide molecules and higher quenching rates [12, 14]. In the next section, we will further explain the dynamic quenching of the AC dye based on quantum theoretical calculations.

### Quenching mechanism based on theoretical calculations

**Fig. 6:**
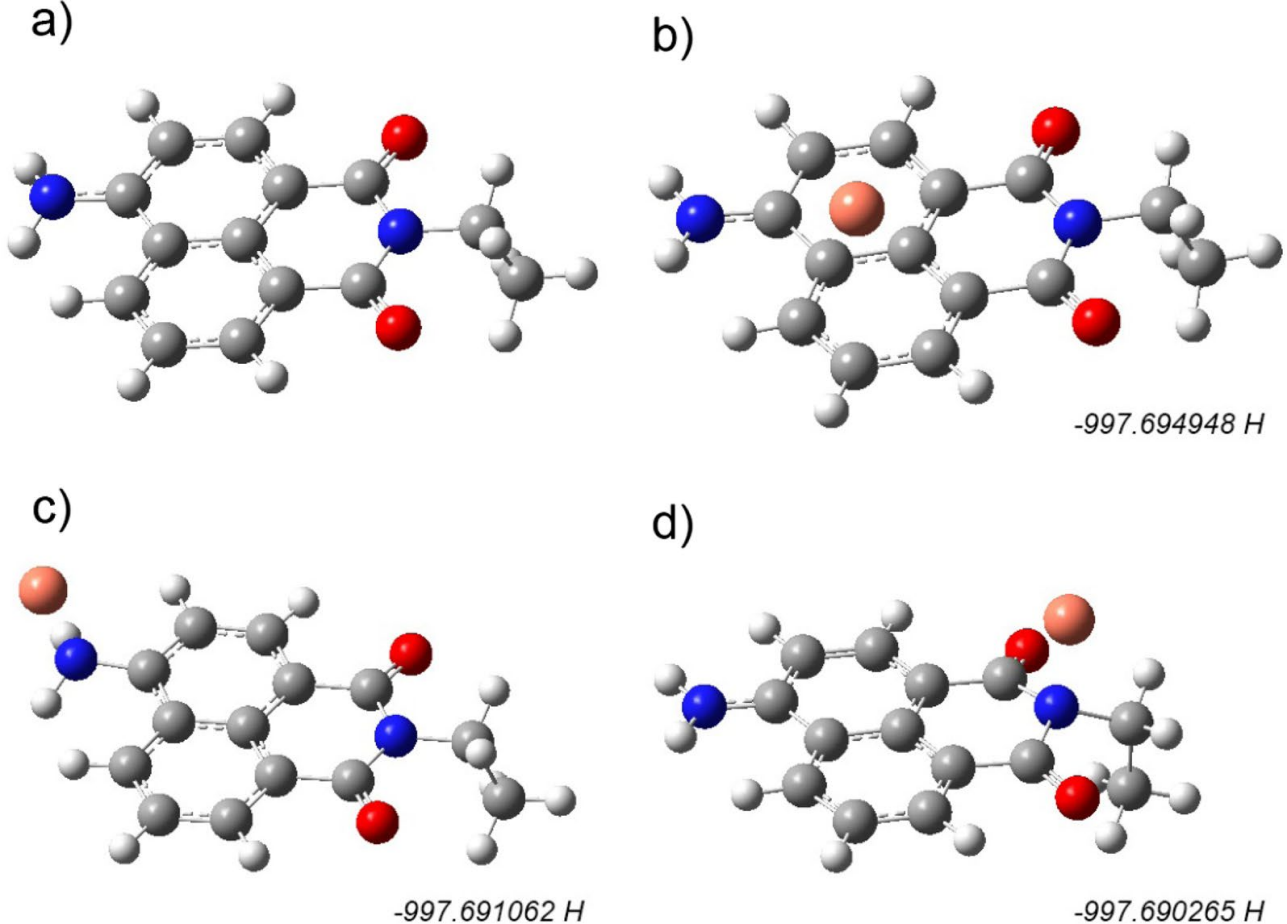
**a** Optimized structure of 4-amino-1,8-naphthalimide (left). **c**, **d** Optimized structures of the naphthalimide with Cu^2+^ ion situated in different positions. The most stable one is the one in **b**. Obtained at B97D3/6–31 + g(d,p) level of theory. Gray = carbon, red = oxygen, blue = nitrogen, white = hydrogen

**Fig. 7:**
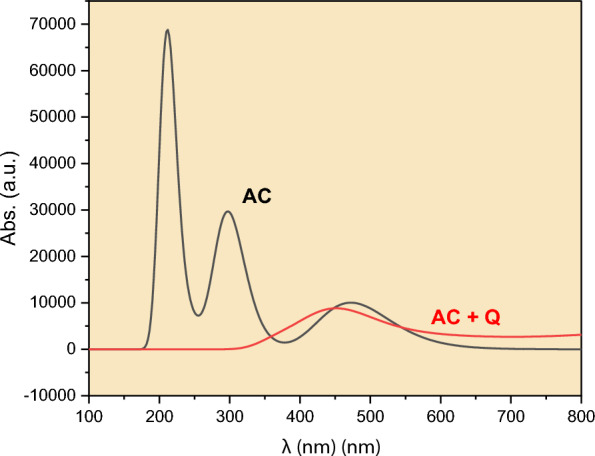
Theoretical absorption spectra of 4-amino-1,8-naphthalimide (black) and with Cu^2+^ ion (red) as obtained by TDDFT calculations at B97D3/6–31 + g(d,p) level of theory

In the previous section, we showed that Cu^2+^ ions are effective quenchers for the AC dye. The quenching was shown to be dynamic based on temperature measurements. To further understand the quenching mechanism, we first optimized the structure of 4-amino-1,8-naphthalimide with and without the Cu^2+^ ion, as shown in Fig. 6a to d. Since the experiments were done using ethyl acetate as a solvent, the optimization was done in the presence of the solvent for more reliability. We tested different geometries for the possible interaction of Cu^2+^ with dye, as illustrated in in Fig. 6b to d. The energy values, in Hartree units, are shown below each figure. In fact, the energy differences between the three geometries is small and lie in the range of 2–10 kJ/mol. The most stable geometry is the one in Fig. 6b, where the Cu^2+^ ion is positioned above the aromatic system. In Fig. 6c, which is only 2.0 kJ/mol higher than its predecessor, the Cu^2+^ is positioned next to the election-rich amine group of the aromatic structure. The N-Cu distance was found to be 2.0 Å, which lies within the range of nitrogen bonded to 3^rd^ raw transition elements. This strong interaction between the naphthalimide and the metal ion has affected the emission spectrum of the dye. Using TDDFT calculations, we obtained the absorption spectra of the two systems as shown in Fig. 7. The black spectrum in the figure represents naphthalimide and it shows a maximum absorbance around 500 nm in accordance with the experiment. After Cu^2+^ was added to the geometry, the absorption spectrum was almost vanished in the 200–400 nm region, as seen from Fig. 7 (red line). The interaction between the Cu^2+^ ion and the dye moiety has resulted in the quenching of the two peaks in the UV region. It is important to note that the excitation occurs in this particular region. Consequently, the presence of Cu^2+^ has influenced the excited state configuration of the dye, thereby hindering its excitation within the 200–400 nm range.

**Fig. 8:**
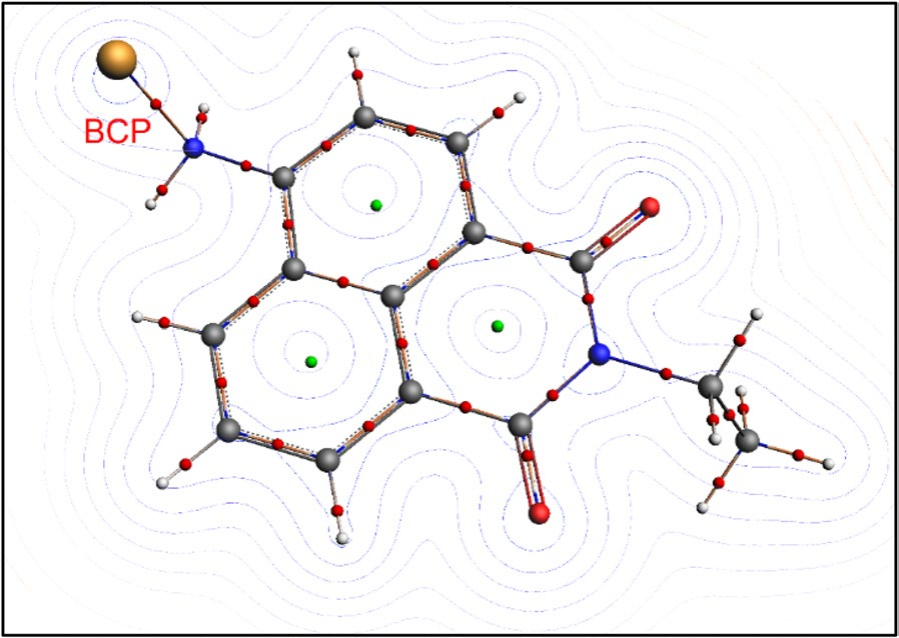
Schematic representation the electron density of 4-amino-1,8-naphthalimide superimposed on the Laplacian contour maps as obtained from QTAIM calculations at B97-D/DZP level of theory. Red dots = BCP, green dots = ring CP

The bond between the Cu atom and the N atom in naphthalimide raises a question about its nature. Hence, we examined the structure using QTAIM calculations. The QTAIM theory, also known as Bader’s theory, uses the topology of the electron density (*ρ*) and its Laplacian (**∇**^*2*^*ρ*) to locate the critical points (CP) in the molecular structures [45, 46]. In addition to *ρ* and **∇**^*2*^*ρ*, QTAIM computes other properties such as the local potential energy density (V), local gradient kinetic energy density (G), and total energy density (H) at each CP. One important type of CPs is those located between non-bonded atoms and are referred to as bond critical points (BCP). The QTAIM properties can then be used to reveal the nature of the BCP [45–47]. The BCP can be classified as either a shared interaction (e.g., covalent and polar) or a closed-shell interaction (e.g., hydrogen bond, ionic, Van der Waals). Figure 8 shows the QTAIM topology of the naphthalimide/Cu^2+^ structure as obtained at B97-D/DZP level of theory. The QTAIM parameters of the nitrogen-copper BCP are listed in Table 1. The electron density of the Cu–N BCP is found to be 0.092 Hartree, which is a typical value for intermolecular interactions (covalent bonds bear higher values). The Laplacian is relatively high and positive (0.397), indicating a VDV interaction, but it still cannot be classified as ionic bond [45, 48]. Another useful criterion is the ratio between the potential (V) and the kinetic energy (G) densities. If the ratio is smaller than 1, the bond is classified as pure closed shell (i.e., ionic, H-bonding, VDV) [48, 49]. Otherwise, a ratio higher than unity refers to a regular closed shell (i.e. covalent). In our case, the V/G is close to unity (1.2), supporting the fact that the N-Cu bond is electrostatic (Van der Waals). In addition, one can obtain the bond energy as it is equal half the value of V_b_ [50]. In our case it is estimated at 185.1 kJ/mol. These facts suggest a strong polar interaction between the Cu^2+^ ion and the naphthalimide moiety, which does not qualify to be covalent or ionic. This strong interaction explains why the fluorescence emission spectrum of the fluorophore decreases when it interacts with the copper (II) quencher. In the previous section, we observed that increasing the temperature resulted in higher quenching. Temperature is unlikely to weaken the strong interaction reflected by a bond energy of 185.1 kJ/mol. Particularly when the temperature difference in our experiments is only 45 °C. As a result, we believe that the quenching is controlled by diffusion, in agreement with the dynamic model. As temperature rises, the quencher diffuses faster in the solution, allowing Cu^2+^ ions to be more accessible.

**Table 1:** Nitrogen-copper BCP topological properties as obtained from QTAIM calculations at B97-D/DZP level of theory

*ρ*_b_(Cu–N) (H)	∇^*2*^*ρ* (H)	− 1/4 ∇^2^(*ρ*) (H)	G_b_(H)	V_b_(H)	H_b_(H)	Bond energy (kJ/mol)	V/G
0.092	0.397	− 0.099	0.120	− 0.141	− 0.021	185.1	1.2

H: Hartree

### Procedure to quantify A/C dye or Cu^2+^ ions using fluorescence spectroscopy

For the future determination of A/C dye’s concentration, one can start by constructing a calibration curve similar to the one in Fig. 2. An analyte solution containing unknown concentration of A/C dye can then be analyzed under the same conditions and its concentration can be determined from the fitting model. As for determining the concentration of Cu^2+^ ions. The procedure is as follows: using a fixed amount of the fluorophore (F_0_), and different standard solutions of Cu^2+^, a quenching curve can be constructed as shown in Fig. 4a. The fluorescence intensity of an analyte containing unknown concentration of Cu^2+^ can be measured in the presence of the same fixed amount of the fluorophore (F_0_). The intensity (F) can then be known from the fitting of the calibration curve (Fig. 2). Finally, using the modified Stern–Volmer model (Eq. 5 and Fig. 4c), the [Cu^2+^] in the analyte sample, which is equal to Q, can be determined.

## Conclusions

The work investigated the use of Cu^2+^ ions as quenchers in naphthalimide based dyes. The work succeeded in developing a rapid, sensitive and accurate technique to quantify both A/C leak detection dye and Cu^2+^ ions, when they are used as a quencher. The results were fitted to a modified SV model and temperature experiments further exhibited the dynamic quenching, as the fluorescence emission spectra was observed to decrease as temperature increased. DFT calculations were conducted to develop a more comprehensive understanding on the mechanism of quenching. As per the QTAIM theory, a strongly polar interaction was found between the Cu^2+^ ion and naphthalimide. This was significant as it led to the conclusion that as per the dynamic model, quenching was controlled strictly by diffusion.

The findings of this work are significant as an efficient analytical system for the quantification of A/C leak detection dyes has been demonstrated. Such dyes may be present in water streams, and consequently be introduced into several food chains. As such, their effect on ecosystems is yet to be declared. Thus, the results of this work may be used to develop a rapid detection method for the presence of any such contaminants. The commercial A/C leak dye used in this work could also be used in further application as a selective chemosensor for the presence of Cu^2+^ ions and serve as a rapid detection method for copper (II) ions.

## Data Availability

The datasets generated during the current study are available in the Mendeley Database repository and can be accessed at: Badran, Ismail (2023), “Dataset for the Fluorescence Quenching of Naphthalimide by Copper (II), Mendeley Data, V1, https://data.mendeley.com/datasets/rdykn2pr6z".
